# Atomic Layer Deposition of Gallium Oxide Films as Gate Dielectrics in AlGaN/GaN Metal–Oxide–Semiconductor High-Electron-Mobility Transistors

**DOI:** 10.1186/s11671-016-1448-z

**Published:** 2016-04-30

**Authors:** Huan-Yu Shih, Fu-Chuan Chu, Atanu Das, Chia-Yu Lee, Ming-Jang Chen, Ray-Ming Lin

**Affiliations:** Department of Material Science and Engineering, National Taiwan University, Taipei, 10617 Taiwan; Department of Electronic Engineering, Chang Gung University, Taoyuan, 333 Taiwan

**Keywords:** GaN, Ga_2_O_3_, Remote plasma atomic layer deposition (RP-ALD), Metal–oxide–semiconductor high-electron-mobility transistor (MOS-HEMT), MOCVD

## Abstract

In this study, films of gallium oxide (Ga_2_O_3_) were prepared through remote plasma atomic layer deposition (RP-ALD) using triethylgallium and oxygen plasma. The chemical composition and optical properties of the Ga_2_O_3_ thin films were investigated; the saturation growth displayed a linear dependence with respect to the number of ALD cycles. These uniform ALD films exhibited excellent uniformity and smooth Ga_2_O_3_–GaN interfaces. An ALD Ga_2_O_3_ film was then used as the gate dielectric and surface passivation layer in a metal–oxide–semiconductor high-electron-mobility transistor (MOS-HEMT), which exhibited device performance superior to that of a corresponding conventional Schottky gate HEMT. Under similar bias conditions, the gate leakage currents of the MOS-HEMT were two orders of magnitude lower than those of the conventional HEMT, with the power-added efficiency enhanced by up to 9 %. The subthreshold swing and effective interfacial state density of the MOS-HEMT were 78 mV decade^–1^ and 3.62 × 10^11^ eV^–1^ cm^–2^, respectively. The direct-current and radio-frequency performances of the MOS-HEMT device were greater than those of the conventional HEMT. In addition, the flicker noise of the MOS-HEMT was lower than that of the conventional HEMT.

## Background

Gallium nitride (GaN)-based semiconductor materials are useful not only in optoelectronic devices but also in millimeter-wave power devices, especially for the fabrication of high-electron-mobility transistors (HEMTs) [1, 2]. For microwave power applications, an AlGaN/GaN HEMT must exhibit high speed, high radio-frequency (RF) power performance, and a high breakdown voltage [3]. Nevertheless, a high gate leakage current is the factor most responsible for limiting the direct-current (DC) and RF power performances of conventional Schottky gate HEMTs [4]. Metal–oxide–semiconductor HEMTs (MOS-HEMTs) can decrease the gate leakage current when incorporating a variety of gate oxide/insulators, including electron beam (EB)-evaporated Pr_2_O_3_ and Er_2_O_3_ [5, 6], thermally oxidized TiO_2_/NiO [7], sputtered Al_2_O_3_ [8], and atomic layer-deposited HfO_2_ and Al_2_O_3_ [9, 10].

Among the established dielectric deposition methods, atomic layer deposition (ALD)—a low-temperature chemical vapor deposition technique in which layer-by-layer deposition occurs based on surface-limited reactions—is attractive because of its accurate control over thickness, excellent step coverage, conformity, high uniformity over large areas, low-defect density, good reproducibility, and low deposition temperatures arising from the self-limiting reactions [11]. These features make ALD a strong candidate for manufacturing nanoscale dielectric layers for electronic devices. Indeed, ALD has been exploited to prepare a variety of high-dielectric-constant (high-*k*) materials (e.g., Al_2_O_3_ [12], HfO_2_ [13], ZrO_2_ [14]) that are used widely in Si-based devices. ALD-deposited high-*k* materials, including HfO_2_, Sc_2_O_3_, and Al_2_O_3_, have been employed as gate dielectric and surface passivation layers to improve the properties of HEMTs [15]. In addition, such binary oxides are thermodynamically stable when they are contacted with III–V semiconductors. Among the high-*k* materials, trivalent Ga_2_O_3_ is a promising material for application as a gate dielectric and passivation layer in III–V semiconductor-based devices because its large band gap (4.9 eV) and moderate dielectric constant (10.6) can help to decrease the leakage current [16]. It was also reported that Ga_2_O_3_ could be a good candidate as a gate dielectric of AlGaN/GaN HEMTs due to the good interface characteristics [17].

Several groups have reported the ALD growth of Ga_2_O_3_. Shan et al. performed thermal ALD of GaN using [(CH_3_)_2_GaNH_2_]_3_ and O_2_ plasma as precursors [18]. In 2012, Comstock and Elam described the ALD of Ga_2_O_3_ films from trimethylgallium and ozone [19]. In 2013, Donmez et al. applied low-temperature ALD to grow Ga_2_O_3_ thin films from trimethylgallium and O_2_ plasma [20]. A temperature window of 100–400 °C has been reported for this process.

In this present study, we prepared high-quality Ga_2_O_3_ thin films through remote plasma atomic layer deposition (RP-ALD) using triethylgallium (TEG) and O_2_ plasma. The remote plasma configuration avoided plasma-induced damage because the wafer was not exposed directly to the plasma, and low-temperature growth mode could realize selective growth by the lift-off method, it made the process much easier and convenient. After investigating the ALD window and characteristics of the Ga_2_O_3_ films, we examined their deposition on AlGaN Schottky layers. Comparing the DC and RF characteristics with those of conventional systems, our proposed ALD Ga_2_O_3_ dielectrics on AlGaN/GaN HEMTs appear to be very promising devices.

## Methods

**Fig. 1:**
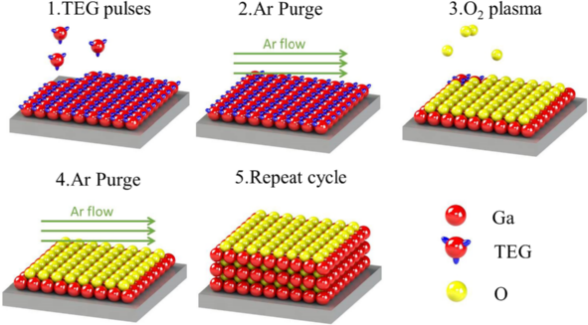
Schematic representation of the ALD process during Ga_2_O_3_ deposition

Ga_2_O_3_ was prepared through remote plasma ALD (Fiji F202, Cambridge Nanotech) using TEG and O_2_ plasma as precursors. The remote O_2_ plasma was generated by an RF coil under an alternative RF power at 300 W. Figure 1 provides a schematic representation of the ALD cycles during the Ga_2_O_3_ deposition process. Each ALD cycle comprised four steps: (1) TEG pulse, (2) Ar purge, (3) O_2_ plasma, and (4) Ar purge. The films were deposited at a temperature of 250 °C with a base pressure of approximately 0.4 Torr.

The thickness and optical characteristics of the Ga_2_O_3_ thin films were measured through spectroscopic ellipsometry (SE, Elli-SE, Ellipso Technology) in the wavelength range 280–980 nm at an incident angle of 70°. The film thickness was confirmed using high-resolution transmission electron microscopy (HRTEM). The chemical compositions and bonding states in the films were characterized using X-ray photoelectron spectrometry (XPS) with Al Kα (1486.6 eV) radiation; pre-sputtering was performed for 10 s to remove any contamination from the surface. The crystal structure of the Ga_2_O_3_ films were characterized by high-power grazing incidence the X-ray diffractometer (GI-XRD; Rigaku TTRAX 3, 18 kW) in θ−2θ mode with Cu Kα radiation. Atomic force microscopy (AFM; Bruker, Edge) was used to evaluate the roughness of the Ga_2_O_3_ surface and interface.

The epitaxial structure was grown on a 2-in silicon carbide substrate using a Nippon Sanso SR-2000 metal-organic chemical vapor deposition system (MOCVD). The epilayer consisted of a 26-nm Al_0.275_Ga_0.725_N barrier layer, a 1-nm AlN inter layer, a 2-μm GaN layer, a 0.7-μm Al_0.07_Ga_0.93_N transition layer, and a 300-nm AlN buffer layer. All epitaxial layers were unintentionally doped. The HEMT structure exhibited a sheet charge density of 1.02 × 10^13^ cm^–2^ and a Hall electron mobility of 1880 cm^2^ V^–1^ s^–1^ at 300 K.

**Fig. 2:**
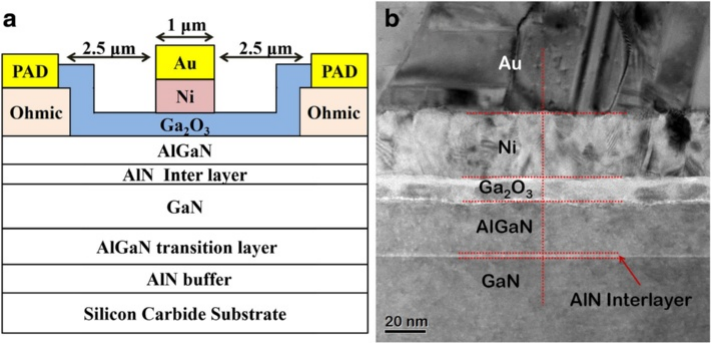
**a** Schematic representation of the cross-sectional structure of a Ga_2_O_3_/AlGaN/AlN/GaN HEMT. **b** Cross-sectional TEM image of a GaN/AlN/AlGaN/Ga_2_O_3_/Ni/Au structure

Devices were processed using conventional optical lithography and lift-off technology. Device isolation was accomplished through mesa dry etching down to the unintentionally doped GaN layer in a BCl_3_ plasma reactive ion etching chamber. Ohmic contacts of Ti/Al/Ni/Au (19/120/30/75 nm) metals were deposited through EB evaporation, followed by rapid thermal annealing at 850 °C for 30 s in a N_2_-rich chamber. After gate lithography pattern formation and surface cleaning, the samples were loaded into the ALD chamber, and a 10-nm Ga_2_O_3_ layer was deposited at 250 °C to function as the gate dielectric and passivation layer between the source and drain contact. Ni/Au (70/140 nm) gate metals were then deposited. For comparison, a conventional Ni/Au Schottky gate AlGaN/GaN HEMT was also fabricated. The Ti/Au (50/1100 nm) metals were deposited as interconnection and probe pads. A schematic cross-sectional structure and a cross-sectional TEM image of a Ga_2_O_3_/AlGaN/AlN/GaN HEMT are presented in Fig. 2a, b, respectively. The gate dimensions of each device were 1 × 100 μm^2^ with a source-to-drain spacing of 6 μm. The microstructures of the fabricated devices were characterized using high-resolution transmission electron microscopy (HRTEM, FEI TecnaiG2 F20). DC characterization of the HEMT devices was performed using an Agilent B1500A semiconductor device analyzer; microwave power measurements were conducted using an ATN load-pull system.

## Results and Discussion

### Characteristics of ALD Ga_2_O_3_

**Fig. 3:**
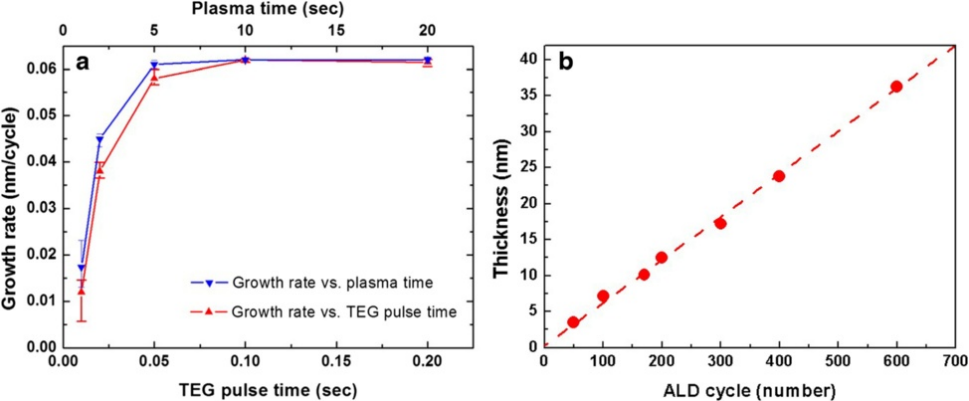
**a** Growth rate of the Ga_2_O_3_ thin films plotted with respect to the TEG pulse time and plasma time. **b** Film thickness plotted with respect to the number of applied ALD cycles

Figure 3a displays the growth rate of the Ga_2_O_3_ thin films as a function of the TEG pulse time and plasma time, at a deposition temperature of 250 °C. The O_2_ flow rate was fixed at 20 sccm. The growth rate is defined here in terms of the film thickness divided by the total number of applied ALD cycles. We observed that the growth rate increased initially upon increasing the TEG dose, but then remained constant at 0.062 nm/cycle when the TEG pulse time was greater than 0.1 s. The growth rate became saturated at plasma times of longer than 5 s. These results suggest that the Ga_2_O_3_ thin films were grown in a self-limiting manner when using the RP-ALD technique. Figure 3b presents the film thickness plotted with respect to the number of applied ALD cycles; the linear dependence implies that the deposition followed the ALD mode and that the film thickness could be control precisely by varying the number of ALD cycles.

**Fig. 4:**
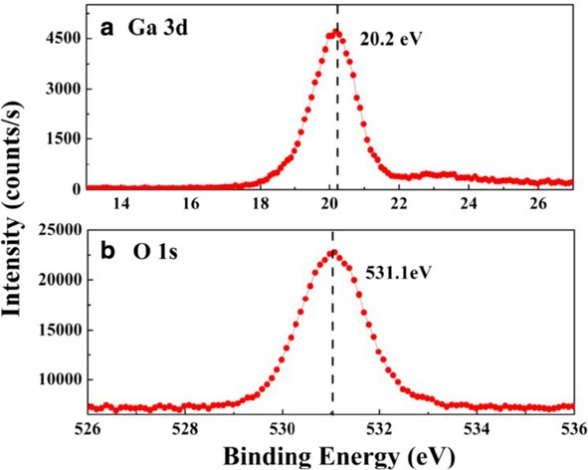
**a** Ga 3d and **b** O 1 s XPS spectra of the Ga_2_O_3_ thin film

Figure 4 displays the XPS spectra of a Ga_2_O_3_ thin film. A single binding energy (BE) peak for the Ga 3d core level, situated at 20.1 eV, confirmed the presence of Ga–O bonds [21] in the sample and the absence of elemental Ga in the film. A single, sharp O 1 s peak, centered at a BE of 531.0 eV, is consistent with previously reported values for the oxide [21]. Taken together, these features confirm that the RP-ALD system facilitated the successful deposition of Ga_2_O_3_ thin films. By measuring relative areas under the curves of the XPS spectra, we calculated average atomic compositions for Ga, O, and C of 41.53, 58.26, and 0.21 %, respectively, in the Ga_2_O_3_ thin film. The molecular ratio of Ga and O in the ALD thin film was slightly higher than ideal (2:3), suggesting the existence of a Ga-rich Ga_2_O_3_ film featuring some O vacancies. The content of carbon atoms was negligible, suggesting that the ethyl groups of TEG had been removed almost completely during exposure to the remote O_2_ plasma.

**Fig. 5:**
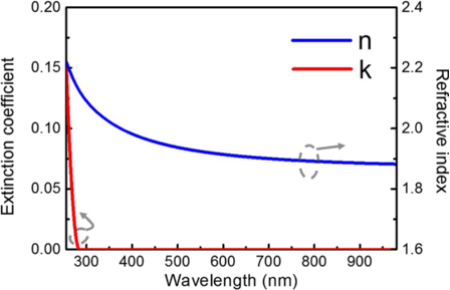
Refractive index and extinction coefficient of the Ga_2_O_3_ thin film plotted with respect to the wavelength

**Fig. 6:**
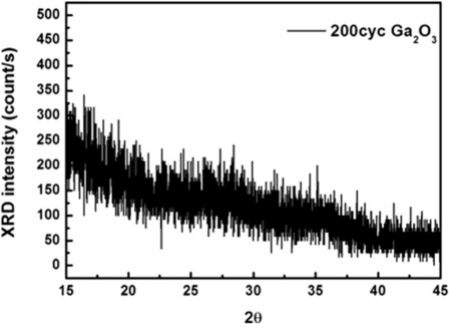
The GI-XRD pattern of the Ga_2_O_3_ films

We used SE to investigate the optical properties of the Ga_2_O_3_ thin film. Figure 5 displays the dispersion of the refractive index and extinction coefficient at wavelengths in the range 280–980 nm. We fitted the SE data to the Tauc–Lorentz model, which is widely used for amorphous semiconductors [22]. The measured refractive index of our ALD Ga_2_O_3_ at a wavelength of 633 nm was 1.91, and its band gap was 4.51 eV; these values are close to those reported [23] for amorphous Ga_2_O_3_. Figure 6 shows the GI-XRD pattern of the Ga_2_O_3_ films. The result was performed with a low-grazing angle of incidence in order to obtain the signal from the thin film. There are no obvious peaks of Ga_2_O_3_ so that the crystal structure was amorphous which was consistent with the SE results.

**Fig. 7:**
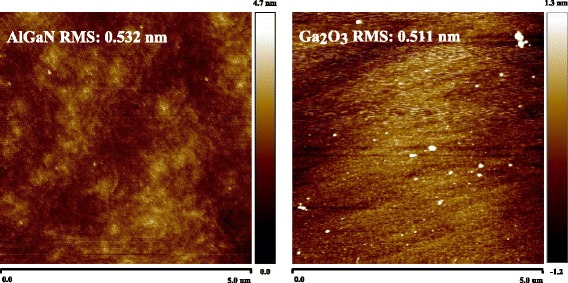
Atomic force microscopy images of the conventional HEMT and the Ga_2_O_3_ MOS-HEMT

To investigate the interface quality, the roughness of the HEMT structure before and after Ga_2_O_3_ grown by ALD were measured by atomic force microscopy (AFM), as shown in Fig. 7. The roughness remained the same order after deposition, this result indicated that the interface between AlGaN and Ga_2_O_3_ should be smooth.

### Characteristics of Ga_2_O_3_ MOS-HEMT

**Fig. 8:**
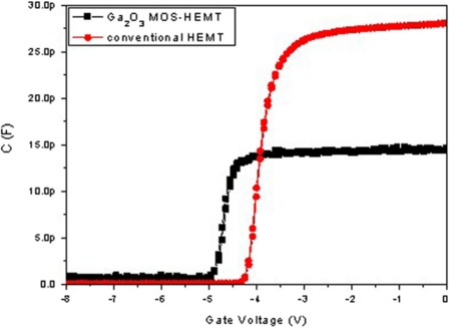
The capacitance-voltage (C-V) characteristics of the conventional HEMT and the Ga_2_O_3_ MOS-HEMT

The capacitance-voltage (C-V) characteristic measured at 1 MHz is shown Fig. 8. The *C*_*ox*_ value come from MOS capacitance; the calculated *C*_*ox*_ values of the conventional HEMT and the Ga_2_O_3_ MOS-HEMT were 28 pF and 14.7 pF, respectively.

**Fig. 9:**
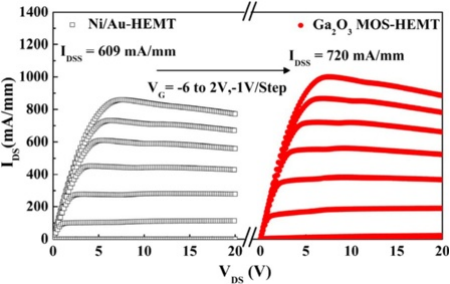
*I*_DS_–*V*_DS_ characteristics of the conventional HEMT and the Ga_2_O_3_ MOS-HEMT upon varying the value of *V*_*G*_ from –6 to +2 V at a step of +1 V

Figure 9 displays the measured *I*_DS_–*V*_DS_ characteristics of the conventional HEMT and the Ga_2_O_3_ MOS-HEMT; they both exhibited good gate modulation and pinch-off characteristics. The measured drain current of the conventional HEMT at a value of *V*_G_ of 0 V (*I*_DSS_) was 609 mA mm^–1^; it was slightly higher (720 mA mm^–1^) for the Ga_2_O_3_ MOS-HEMT.

**Fig. 10:**
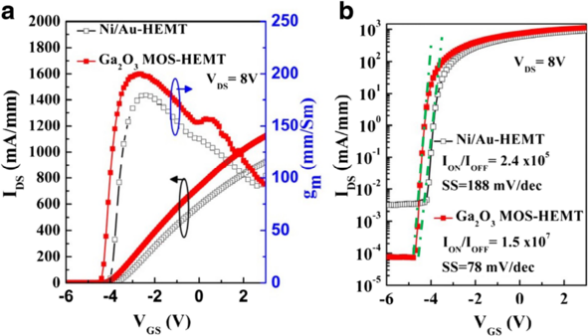
**a** Transfer characteristics and **b** log *I*_DS_ vs *V*_GS_ plots of the conventional HEMT and the Ga_2_O_3_ MOS-HEMT

Figure 10a presents the transconductance (*g*_m_) and drain current (*I*_DS_) characteristics for these devices. The maximum transconductances (*g*_m max_) of the conventional HEMT and Ga_2_O_3_ MOS-HEMT when biased at a value of *V*_DS_ of 8 V were 179 and 200 mS mm^–1^, respectively; their maximum drain currents (*I*_DS max_) were 921 and 1123 mA mm^–1^, respectively. Thus, the values of *I*_DS max_ and *g*_m max_ of the MOS-HEMT were relatively high, presumably the result of enhanced mobility caused by decreased carrier scattering, due to surface passivation [24, 25]. In addition, the slight increase in the gate-to-channel separation, resulting from the presence of the Ga_2_O_3_ gate oxide layer, was responsible for the threshold voltage shifting from –3.8 to –4.2 V. For the HEMT with Ga_2_O_3_, the threshold voltage (*V*_th_) shift, which was generally resulted from the defects in interface and gate oxide, was smaller than that with Al_2_O_3_ [26] and HfO_2_ [27]. The result may be caused by the excellent interface between Ga_2_O_3_ and AlGaN, optimized RP-ALD process condition, and the use of TEG (reduce defects in Ga_2_O_3_). To further investigate the gate control characteristics of both devices, we studied the region near the cut-off voltage. The subthreshold swing (SS) is a parameter that indicates how effectively a device can be turned off; it is defined as the decrease in the log (*I*_DS_)–*V*_GS_ plot near the cut-off voltage as shown in Fig. 10b. We measured the values of *I*_DS_ with respect to *V*_*GS*_ for both devices biased at a value of *V*_DS_ of 8 V. The *I*_ON_/*I*_OFF_ ratio and SS of the Ga_2_O_3_ MOS-HEMT (1.5 × 10^7^ and 78 mV decade^–1^, respectively) were superior to those of the conventional HEMT (2.4 × 10^5^ and 188 mV decade^–1^, respectively). Figure 10b also displays the values of the OFF-state *I*_DS_, revealing that the leakage current (3.3 × 10^–3^ mA mm^–1^) of the conventional HEMT decreased (to 7.45 × 10^–5^ mA mm^–1^) after deposition of the Ga_2_O_3_ thin layer. We calculated the effective interfacial state density from the SS [6]. By neglecting the depletion capacitance in the active layer, the value of *N*_t_ can be estimated as

1 $$ {N}_t=\left(\frac{SS}{ln10}\cdot \frac{q}{KT}-1\right)\frac{C_{ox}}{q}, $$

where *K* is the Boltzmann constant, *T* is the temperature, *C*_*ox*_ is the capacitance of the gate oxide and *q* is the quantity of one electron, respectively. The effective interfacial states density (4.77 × 10^12^ eV^–1^ cm^–2^) of the conventional HEMT decreased to 3.62 × 10^11^ eV^–1^ cm^–2^ for the Ga_2_O_3_ MOS-HEMT.

**Fig. 11:**
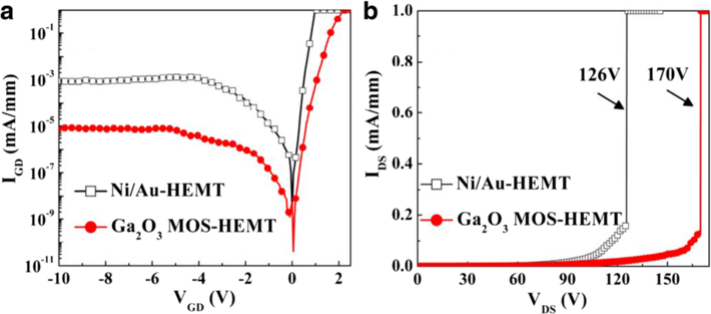
**a** Gate leakage current and **b** three-terminal off-state breakdown characteristics of the conventional HEMT and the Ga_2_O_3_ MOS-HEMT

Figure 11a presents the gate leakage current densities of the two devices. Under reverse bias conditions, the leakage current density of the Ga_2_O_3_ MOS-HEMT reached as low as 7.8 × 10^–6^ mA mm^–1^ at a value of *V*_GD_ of –10 V; this leakage current density is two order of magnitude lower than that of the conventional HEMT (8.59 × 10^–4^ mA mm^–1^). Figure 11b displays the three-terminal off-state breakdown characteristics of the conventional HEMT and Ga_2_O_3_ MOS-HEMT measured at a value of *V*_GS_ of –6 V. The drain breakdown voltage of the Ga_2_O_3_ MOS-HEMT (170 V) was 44 V larger than that of the conventional HEMT fabricated on the same wafer. The high quality of the ALD Ga_2_O_3_ film effectively suppressed the gate leakage current density and improved the breakdown voltage because of its large band gap [16].

**Fig. 12:**
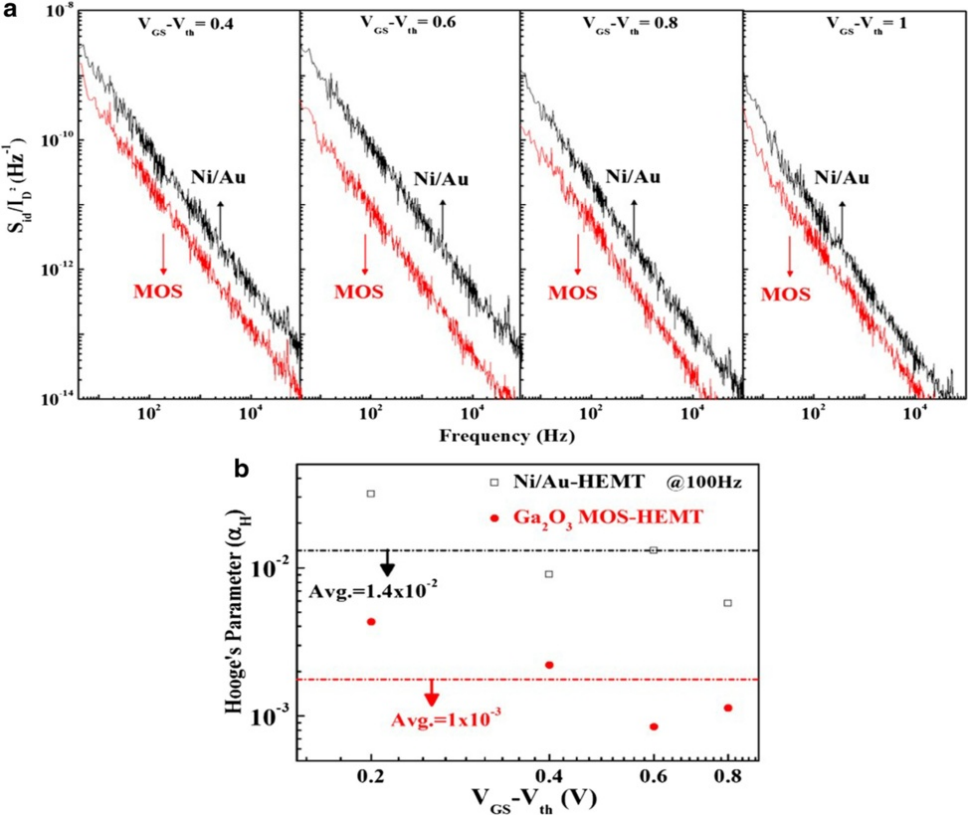
**a** Normalized values of *S*_ID_/*I*_D_^2^ plotted at various values of (*V*_GS_–*V*_th_); **b** Hooge’s coefficient (*α*_H_) plotted with respect to (*V*_GS_–*V*_th_) for the conventional HEMT and the Ga_2_O_3_ MOS-HEMT

We conducted 1/*f* noise measurements to elucidate the relationship between the low-frequency noise and the gate electrode interface; here, we varied the frequency from 1 to 100 KHz and the gate overdrive bias (*V*_GS_–*V*_th_) from 0.4 to 1 V in steps of 0.2 V. Figure 12a displays the gate bias dependence of the normalized drain current noise spectral density (*S*_ID_/*I*_D_^2^) of both devices at a value of *V*_DS_ of 2 V. The 1/*f* noise spectrum of the Ga_2_O_3_ MOS-HEMT was lower than that of the conventional HEMT, due to its lower gate leakage current.

Our findings indicate that lower interfacial states can be achieved when using this ALD-deposited Ga_2_O_3_ film as a gate dielectric and passivation layer. Furthermore, Hooge’s coefficient (*αH*) is another noise parameter that quantifies the 1/*f* noise; it can provide a measure of the total number of active traps causing the noise and can be used as a rough figure of merit for both devices. Hooge’s coefficient can be expressed as follows [28]:

2 $$ \alpha H=\left(\frac{fWL{C}_i\left({V}_{\mathrm{GS}}-{V}_{\mathrm{th}}\right){S}_{\mathrm{ID}}}{q{I}^2D}\right), $$

where *f* is the measurement frequency, *C*_*i*_ is the unit capacitance of the gate material, and *q* is the elementary electron charge.

Figure 12b presents the values of *αH* plotted with respect to (*V*_GS_–*V*_th_), measured at a value of *f* of 100 Hz. The average values of *αH* for the conventional HEMT and Ga_2_O_3_ MOS-HEMT were 1.4 × 10^–2^ and 1 × 10^–3^, respectively. The flicker noise spectral density of the Ga_2_O_3_ MOS-HEMT was lower than that of the conventional HEMT because of its lower number of interfacial states.

**Fig. 13:**
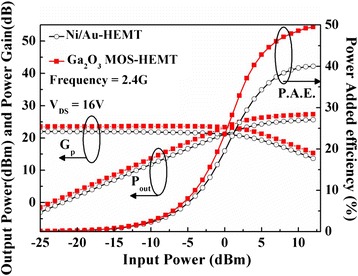
Microwave power characteristics of the conventional HEMT and the Ga_2_O_3_ MOS-HEMT

Figure 13 displays the microwave output power (*P*_out_), power gain (*G*_p_), and power-added efficiency (PAE) characteristics for both devices determined at 2.4 GHz with a drain bias of 16 V, measured on-wafer by the load-pull ATN system with automatic tuners measuring the optimum-load impedance for maximum output power. The conventional HEMT exhibited an output power of 25.6 dBm; the associated power-added efficiency was 40 % and the power gain was 21.9 dB. For the Ga_2_O_3_ MOS-HEMT, the output power was 27.3 dBm; the associated power-added efficiency was 49 % and the power gain was 23.6 dB. Output power and PAE can be expressed as

3 $$ {P}_{\mathrm{out}}=\frac{1}{8}\left({V}_{\mathrm{DS}}-{V}_{\mathrm{Kness}}\right)\times {I}_{DS} $$

and

4 $$ \mathrm{P}\mathrm{A}\mathrm{E}=\frac{P_{\mathrm{out}}-{P}_{\mathrm{in}}}{P_{\mathrm{DC}}}\times 100\%, $$

where *V*_knee_ is the knee voltage, *P*_out_ is the output power, *P*_in_ is the input power, and *P*_DC_ is the DC power supply. The relatively high RF power performance of the Ga_2_O_3_ MOS-HEMT resulted from its higher current drive, lower *P*_DC_ dissipation, and lower gate leakage current, all arising from the good passivation and gate insulation effects of the Ga_2_O_3_ film prepared through remote plasma ALD [5, 25, 29].

## Conclusions

We have used remote plasma ALD to deposit Ga_2_O_3_ films that we then applied in AlGaN/AlN/GaN HEMTs on silicon carbide substrates. The thin Ga_2_O_3_ films prepared through RP-ALD exhibited saturation of the growth rate upon increasing the TEG pulse time and plasma time. The film thickness varied linearly with respect to the number of ALD cycles. This behavior is consistent with the growth of Ga_2_O_3_ following the ALD mode. The ALD Ga_2_O_3_ films possessed excellent uniformity and the Ga_2_O_3_–GaN interfaces were smooth. The fabricated Ga_2_O_3_ MOS-HEMT exhibited enhanced gate insulating and surface passivation effects, resulting in superior DC and RF performance relative to those of the conventional HEMT. Moreover, the low leakage current and low interfacial state density of the Ga_2_O_3_ MOS-HEMT provided a measured SS of 78 mV decade^–1^ and an *I*_ON_/*I*_OFF_ ratio that was greater than 10^7^ times that of the conventional HEMT. These attractive features of the HEMT incorporating the ALD-prepared Ga_2_O_3_ gate dielectric suggest that ALD-prepared Ga_2_O_3_ might find further applicability in other high-power devices in the near future.
